# Perineural invasion and perineural spread in periocular squamous cell carcinoma

**DOI:** 10.1038/s41433-022-02306-w

**Published:** 2022-11-18

**Authors:** Jessica Y. Tong, Shyamala C. Huilgol, Craig James, Saul Rajak, Dinesh Selva

**Affiliations:** 1 South Australian Institute of Ophthalmology, Royal Adelaide Hospital, Adelaide, SA Australia; 2 Save Sight Institute, Sydney Medical School, Faculty of Medicine and Health, The University of Sydney, Sydney, NSW Australia; 3 Adelaide Skin & Eye Centre, Kent Town, SA Australia; 4 Department of Dermatology, Royal Adelaide Hospital, The University of Adelaide, Adelaide, SA Australia; 5 Clinpath Laboratories, Adelaide, SA Australia; 6 The Sussex Eye Hospital, University Hospitals Sussex, Brighton, UK

**Keywords:** Eye cancer, Eyelid diseases

## Abstract

Perineural invasion (PNI) in cutaneous squamous cell carcinoma (SCC) of the periocular region is a prognostic marker of adverse tumour outcomes. PNI carries a well-established risk of tumour recurrence, regional metastasis and higher likelihood of mortality. This review will explore and stratify the risks conferred by histological PNI parameters. The radiological features of perineural spread (PNS) and the imaging sequences that delineate these findings will also be highlighted. Surgical excision with en face margin control is the preferred technique for achieving histological clearance. Adjuvant radiotherapy improves treatment outcomes in the setting of concomitant high-risk features. For locally advanced or metastatic cutaneous SCC, immunotherapy represents a novel treatment alternative. This review will provide an algorithm for the diagnosis and management of periocular SCC with PNI and PNS.

## Abstract (zh)

眼周皮肤鳞状细胞癌 (SCC) 的周围神经浸润 (PNI) 是恶性肿瘤结局的预后标志。目前公认为PNI的出现具有肿瘤复发, 区域转移和高死亡率的风险。本综述将探讨并分层说明组织学PNI参数带来的风险。此外还重点介绍神经周围扩散 (PNS) 的放射学特征和描述这些结果的成像序列。手术切除联合en face控制边缘是实现组织学清除的首选技术。伴随高危特征的条件下, 辅助性放疗可改善治疗效果。对于局部晚期或转移性的 SCC, 免疫疗法代表了一种新型的治疗手段。本综述将为伴有PNI和PNS的SCC提供一个诊断和管理的算法。

## Introduction

Squamous cell carcinoma (SCC) is the second most common cutaneous malignancy of the eyelid and periorbital region in the West, while large epidemiological studies from India and China have shown that it is the third most prevalent malignant eyelid tumour [1–3]. SCC is characterised by its potential for rapid growth, deep subclinical extension and propensity for perineural and vascular invasion [1]. The potential sequelae of periocular SCC include orbital and intracranial invasion, nodal and distant metastasis, and mortality.

Perineural invasion (PNI) is a histological marker of aggressive disease that is associated with locoregional recurrence, metastasis and reduced tumour-specific survival [1, 4]. It often occurs concurrently with other high-risk features including large tumour size, poor histological differentiation, lymphovascular invasion and chronic immunosuppression [5]. The incidence of PNI is reported to be 2.5–14.0% of head and neck SCC [4, 6, 7], and 4.3–14.4% of periocular SCC [8–12]. Other studies of eyelid SCCs have reported a higher incidence of PNI ranging from 23.8 to 36.7%, which may be explained by more advanced disease at presentation [13–15].

It is important to understand the distinction between the terms PNI and perineural spread (PNS) as the latter confers a different prognosis and management paradigm. PNI is a histological term that refers to findings within a resection specimen. PNS refers to clinically manifest symptoms of pain or dysesthesia, and/or radiologically detectable perineural infiltration. PNS has also been termed ‘clinical/radiological PNI’ which may be a source of confusion and, hence, in this review we will use the terms PNI and PNS as defined above in concordance with the current literature.

It should be noted that while there is good evidence for the prognostic implications of PNI/PNS, there is a lack of robust randomised controlled trials to provide definitive management guidelines. Furthermore, the majority of evidence is derived from head and neck cSCC rather than the relatively sparse periocular SCC literature which is mostly comprised of retrospective studies.

This review aims to provide clinicians with a pragmatic approach to the risk stratification and management of periocular SCC with PNI and PNS. Histological features of PNI and their prognostication will be evaluated. En face margin control techniques are preferable to confirm histological clearance. Adjuvant radiotherapy is warranted in the presence of concomitant high-risk histological and clinical features. The clinico-radiological characteristics and management implications of PNS are also reviewed

## Pathogenesis of PNI

The peripheral nerve consists of three layers: (1) the innermost endoneurium encircling axons, (2) the middle perineurium surrounding fascicles, and (3) the outermost epineurium which binds fascicles to form a peripheral nerve [16]. The perineurium forms a mechanical barrier against toxins with multiple concentric layers of endothelial cells bound by tight junctions, while the endoneurium is supported by a blood-nerve barrier [17]. These innate defence mechanisms refute the notion of a low-resistance plane within the perineural space. The neurotrophic behaviour of SCCs is likely driven by alternative processes.

At a cellular level, SCCs may remodel the peri-tumoral environment via neurotrophic growth factor receptors (tyrosine receptor kinase A, B, C) and nerve cell adhesion molecule (N-CAM), which facilitate the attachment and invasion of tumour cells beyond the perineural sheath [18, 19]. Deposition of laminin-5, a basement membrane protein, also plays a role in the modulation of cellular adhesion and inflammatory cascade signalling, thereby enabling invasion into the perineural space [20]. There is a significant association between programmed death ligand 1 (PD-L1) expression on immunohistochemical staining and pathological features with a predilection for metastasis (namely large nerve diameter, poor differentiation and tumour thickness) [21].

## Histological features of PNI

### Structural changes in PNI

**Fig. 1:**
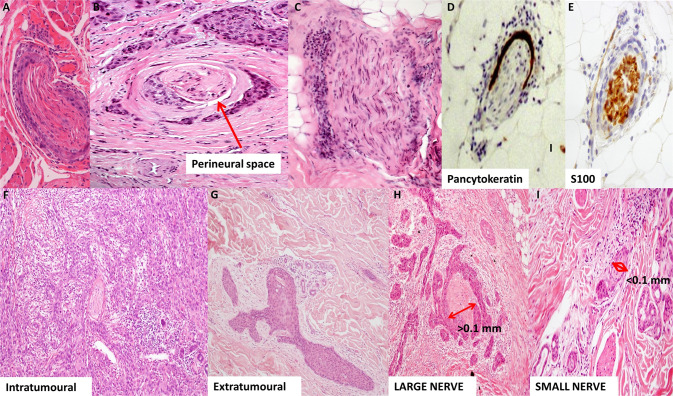
Histological findings in perineural invasion (PNI). **A**, **B** Haematoxylin & eosin stain demonstrating invasion of tumour cells into the perineural space involving at least one-third of the nerve circumference. **C** Perineural lymphocytic inflammation is characteristic of adjacent PNI. **D**, **E** Immunohistochemical staining for pancytokeratin (carcinoma) and S100 (nerve) improves the detection of PNI. **F** Intratumoral PNI is defined as an involved nerve within the bulk of the tumour mass. **G** Extratumoral PNI describes nerve involvement that is distant from the main tumour mass. **H**, **I** Large-calibre and small-calibre nerves are distinguished by a threshold of 0.1 mm in nerve diameter.

PNI is characterised by microscopic tumour invasion into the space between or beneath the interdigitating perineural layers, involving at least one-third of the nerve circumference (Fig. 1A, B) [16, 22, 23]. The nerve itself undergoes structural deterioration with axonal and myelin damage, chronic inflammation and fibrosis [24]. In the absence of an overtly involved nerve, signs of perineural inflammation with lymphocytes (Fig. 1C) may represent residual adjacent tumour [7, 25]. The use of pancytokeratin and S100 immunostaining with multiple examined levels may also improve detection of subtle PNI (Fig. 1D, E) [26]. The absence of demonstrable nerves in the dermis may also reflect more proximal PNI with Wallerian degeneration of the distal smaller cutaneous nerves [27].

### Extratumoral vs intratumoral PNI

The location of PNI relative to the tumour mass has prognostic significance. Intratumoral PNI describes invasion within the bulk of the tumour mass, which appears to have little relevance regarding tumour outcomes (Fig. 1F). On the contrary, extratumoral involvement refers to PNI beyond the main tumour mass (Fig. 1G); it is characteristic of more aggressive lesions, particularly in the presence of established orbital signs [1]. There are no comparative studies in the periocular SCC literature to define outcomes in each division, but there is convincing evidence from head and neck non-cutaneous SCC studies that extratumoral PNI is associated with worse disease-free survival outcomes [28, 29].

### Focal vs extensive PNI

PNI can be further subdivided into ‘focal’ involvement with 1–2 positive nerves, or ‘extensive’ involvement of greater than 2 nerves on the histological field [30]. Focal PNI is associated with a better 5-year relapse-free survival rate than extensive nerve involvement [31].

### Skip lesions

Cutaneous SCC (cSCC) has been previously associated with so-called perineural skip lesions [32]. Therefore, supposedly clear resection margins may miss more proximal disease. The uncertainty of residual disease has prompted some clinicians to deliver radiotherapy despite clear surgical margins [33, 34]. However, ‘skip lesion’ is considered an erroneous term and may in fact represent processing artefacts, perineural inflammation obscuring true PNI, and focal regression [16, 35]. Panizza et al. found that tumours only spread contiguously along a peripheral nerve and therefore radiotherapy should not be unnecessarily extended to encompass the possibility of skip lesions [16, 36].

### Nerve calibre

PNI with large-calibre involvement (≥0.1 mm in diameter) is a poor prognostic marker in cSCC and significantly associated with other high-risk histological characteristics. Large-calibre PNI (Fig. 1H) is more likely to be extratumoral, deep (invasion beyond the subcutaneous fat), and have multiple nerve involvement [26, 37]. Furthermore, it is associated with lymphatic progression, nodal metastasis and increased risk of disease-related death compared with small-calibre PNI (Fig. 1I) [26, 38–40].

Histological parameters of PNI have been evaluated to determine if there is a spectrum of PNI ‘severity’ [41]. These parameters were nerve calibre (diameter of largest nerve involved), multiplicity and depth of nerve involvement, and location (intratumoral versus extratumoral; focal versus circumferential) [41]. Of these features, only nerve diameter and the number of nerves were independently associated with local recurrence, metastasis or tumour-related death. The authors reported that nerve calibre ≥0.2 mm diameter conferred a higher risk of adverse outcomes. This suggests that beyond the 0.1 mm threshold, the risk increases proportionally with greater nerve diameter involvement [41].

### Depth of PNI

In a separate multicentre retrospective study of cSCC, depth of nerve involvement was a significant factor associated with lymphatic progression and disease-specific death in univariate analyses. Although deep nerve involvement lost statistical significance on multivariate calculations, it commonly occurs in the presence of other high-risk tumour factors and should nonetheless warrant caution [38].

## Defining high-risk PNI

PNI in cSCC is ubiquitously considered a poor prognostic risk factor in international cancer guidelines [42–45]. However, the literature suggests that a more nuanced approach can be applied to further stratify PNI parameters into a very high-risk category. This includes extensive PNI (involvement of >2 nerves on a histological field), named nerve or large-calibre (≥0.1 mm) involvement, intra- vs extratumoral involvement and deep invasion beyond the subcutaneous fat (Table 1).

**Table 1:** Risk stratification of perineural invasion in periocular SCC.

Low risk	High risk	Very high risk
No PNI	PNI present	PNI present
	∙ Periocular region	∙ Extratumoral involvement
	∙ Focal involvement	∙ Extensive involvement
	∙ Small-calibre nerve <0.1 mm	∙ Named nerve or large-calibre ≥0.1 mm
	∙ Dermal invasion only	∙ Deep invasion beyond subcutaneous fat

## Additional high-risk tumour factors

Although this review focus is on PNI, it would be remiss not to highlight other parameters that increase the risk of developing PNI and individually contribute to an aggressive tumour profile. A summary of these high-risk parameters is provided in Table 2.

- The Australian Cancer Council (2019) and American National Comprehensive Cancer Network (NCCN) Version1.2022 guidelines recognise the periorbital region and eyelid as high-risk locations for overall prognosis in cSCC [46, 47].
- Tumour size greater than 2 cm and a higher AJCC T category (defined by tumour size and degree of eyelid margin involvement) are both important determinants of PNI risk [10, 43, 48].
- For periocular SCC, longer duration of the lesion is associated with a higher risk of orbital invasion and PNI [14].
- PNI is significantly more common in recurrent SCC than primary tumours. Furthermore, lesions that have undergone a greater number of surgical excisions, indicative of recurrent or aggressive disease, are more likely to have PNI [48].
- Aggressive SCC phenotypes with atypical histology (spindle cell, adenosquamous and post radiotherapy variants) and moderate to poor differentiation are associated with a higher incidence of PNI [4, 48]. The desmoplastic subtype of SCC significantly increases the risk of orbital invasion [49].
- Tumour thickness greater than 6 mm is associated with higher risk of recurrence and metastasis [50].
- Other risk factors identified for nodal metastasis in head and neck cSCC are lymphovascular invasion, poorly differentiated histology and chronic immunosuppression [10, 13, 51]. There is scant data on their prognostication in periocular SCC, but it is reasonable to extrapolate and postulate that these risk factors likely play a similar role.
- The 40-gene expression profiling test is a newly validated assessment that identifies cSCC lesions as low, high or very high-risk, of which the latter group has a ≥50% metastatic risk. Appropriate risk stratification enables judicious use of resources for aggressive surveillance and high-intensity management strategies [52].

**Table 2:** Summary of clinical and histological risk factors in periocular SCC.

	Low risk	High risk	Very high risk
Clinical factors	Depth of invasion <2 mm	Periocular location Recurrent tumour	Tumour size ≥2 cm Tumour thickness >6 mm Chronic immunosuppression
Histological factors	Well differentiated No PNI	Moderately differentiated Acantholytic, adenosquamous or metaplastic subtype	Poorly differentiated Desmoplastic subtype Post radiation carcinoma PNI Lymphovascular invasion

## Perineural spread (PNS)

PNS predominantly affects the trigeminal and facial nerves due to an extensive plexus of subcutaneous nerves [1, 4, 53]. It usually occurs in a retrograde fashion [16]. The distribution is skewed to the trigeminal nerve (V1 alone 24.3%, V2 alone 28.8% and V3 alone 4.5%) compared with sole facial nerve involvement (5.4%) or combined trigemino-facial spread (8.1%) [54]. Around the periocular region, there are numerous trigemino-facial anastomoses with connections between: (a) the infraorbital nerve and the zygomatic branch of the facial nerve, (b) the supraorbital nerve with the temporal branch of the facial nerve, and (c) the zygomaticofacial branch of the maxillary nerve and the zygomatic branch of the facial nerve [55].

## Clinical features of PNS

The median time between onset of primary cSCC and PNS is 16 months [56]. A summary of common symptoms and their frequency is outlined in Table 3. Pain is common but early symptoms include paraesthesia, hypoesthesia, and formication which is the sensation of insects crawling across one’s skin [14, 57]. Motor deficits are variable, but complete ophthalmoplegia is highly suggestive of orbital apex involvement [57, 58]. A lower motor neuron facial nerve palsy with progressive paralysis, absence of functional recovery after 6 months and facial hyperkinesia should prompt suspicion for neoplastic infiltration [17, 53, 59].

**Table 3:** Clinical features of perineural spread.

Clinical features	Frequency (%)
Numbness	56.7
Neuropathic pain	43.3
Paraesthesia	30.8
Formication	15.0
Burning	3.3
Subcutaneous mass	34.2
Facial palsy	39.2
Partial palsy	59.6
Complete palsy	40.4
Progressive onset	89.5
Sudden onset	10.5

Adapted from Warren et al. [54].

Orbital SCC associated with PNS can occasionally present with a subcutaneous or intraorbital mass in the distribution of the involved nerve. These masses may have a significant cystic component [24, 58, 60]. A subcutaneous nodule in the cheek, forehead or frontal scalp may represent infraorbital or frontal nerve involvement, respectively [61]. Intraorbital PNS most commonly develops in the superomedial orbit as an enlarged cord at the supraorbital foramen and can extend posteriorly, resulting in globe dystopia and orbital apex syndrome [24, 57].

## Radiological features of PNS

### Magnetic resonance imaging

On MRI, PNS is characterised by asymmetrical thickening and enhancement of the involved nerve [62, 63]. Occasionally, PNS to the orbit can manifest as a cystic mass demonstrating an isointense T1 signal, hyperintense T2 signal and circumferential enhancement [24]. Physiological enhancement of the maxillary, mandibular and facial nerves can be visualised as a ‘target’ sign or ‘tram track’ pattern at the foramen rotundum, foramen ovale and stylomastoid foramen, respectively [64, 65]. Tumour infiltration may lead to heterogeneously affected segments, giving rise to a ‘string of beads’ appearance [64]. Neural enhancement occurs due to perineural inflammation, demyelination, ischaemia and/or axonal degeneration. Disruption of the perineural tight junctions and endoneurial blood-nerve barrier leads to extravasation of contrast material into the nerve substance [66]. Enhancement is therefore an earlier radiological sign than nerve enlargement [67]. Progressive and often concentric neural enlargement will cause secondary erosion and widening of the neural foramina at the midface and skull base [55, 64]. It should be noted that despite treatment response, there is often persistent perineural thickening or enhancement [68]. Therefore, on imaging surveillance, stabilisation or regression of the signal without further nerve enlargement represents disease control [64].

On T1-weighted sequences, effacement of fat pads at the skull base foramina is an important early sign of PNS. Fat signal alterations may be seen along the course of the ophthalmic nerve at the superior orbital fissure, within the orbital roof superior to the superior rectus/levator complex and at the supraorbital foramen. Changes in the peri-antral fat pads near the infraorbital foramen, foramen rotundum and pterygopalatine fossa may indicate PNS along the maxillary nerve and its infraorbital branch. Fat density changes at the stylomastoid foramen are indicative of facial nerve involvement [55].

**Fig. 2:**
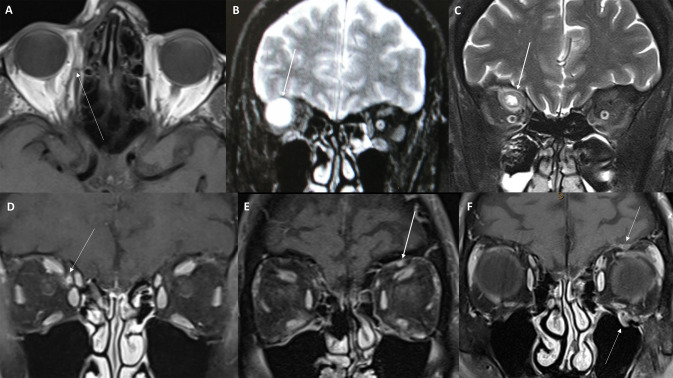
MRI findings in periocular squamous cell carcinoma with perineural spread. **A** Axial T1-weighted sequence demonstrates thickening of the right nasociliary nerve and anterior ethmoidal nerve (arrow). **B** Perineural spread in the orbit can manifest as a cystic mass with a hyperintense T2 signal (arrow), which may be representative of hypercellularity secondary to neoplastic infiltration. **C** T2 coronal sequence demonstrating right frontal nerve enhancement and enlargement. **D**–**F** Coronal T1 fat-suppressed contrast-enhanced sequences demonstrating different examples of V1 and V2 perineural spread with enlargement and enhancement of the nasociliary nerve (**D**), frontal nerve (**E**, **F**) and infraorbital nerve (**F**).

High-resolution MRI performed with a dedicated field of view and smaller slice thickness is the imaging modality of choice for visualising PNS [62]. Examples of PNS secondary to periocular SCC are provided in Fig. 2. A summary of the pertinent MRI features and recommended imaging protocols is provided in Table 4.

- To detect PNS to the trigeminal and facial nerve branches, a targeted field of view on axial slices should extend from the frontal sinus to the hyoid, and on coronal sections, it should encompass the nasal ala, anterior orbital border and the pons.
- T1WI is the sequence best placed to visualise effacement of fat pads along the course of involved nerves and at the foramina. Contrast-enhanced T1WI with fat suppression enables the evaluation of nerve enlargement and enhancement. T2WI with fat saturation can detect denervation and secondary atrophy of the muscles of mastication and facial expression. Affected muscles demonstrate a high T2 signal and with time, adipogenic replacement will be evident on non-fat-suppressed sequences [64, 67].
- Apparent diffusion coefficient (ADC) has emerged as a useful quantitative measure of the diffusion weighted imaging sequence. Restricted diffusion, and therefore a lower ADC value, may be representative of cytotoxic oedema and hypercellularity from neoplastic infiltration of an involved nerve [55, 69]. There is no robust evidence to establish definitive associations between restricted diffusion and PNS, but it may provide useful ancillary data in the future.

**Table 4:** Radiological features of perineural spread and the recommended sequences to enable detection of these findings.

MRI features	MRI PNI protocol
∙ Nerve thickening or enlargement ∙ Nerve enhancement ∙ Obliteration of fat pads ∘ V1: supraorbital foramen, orbital roof superior to levator aponeurosis, superior orbital fissure and orbital apex ∘ V2: pterygopalatine fossa ∘ VII: stylomastoid foramen ∙ Erosion and widening of the bony foramina ∙ Muscle denervation atrophy and adipogenic replacement	MR imaging skull base and face: CN I–VII ∙ Hyoid to frontal sinus: axial T1 noncontrast 2.5 mm, axial T2 fat-saturated 512/3 mm ∙ Nasal ala to pons: coronal T1 noncontrast 2.5 mm, coronal 3D T2 0.8 mm ∙ Axial and coronal T1 fat-saturated contrast-enhanced, multiplanar reconstruction 1 mm

Adapted from Schuknecht [64].

### CT and CT-PET imaging

Computed tomography has a limited role for detection of PNS. Although enlargement of neural foramina can be visualised on bone windows, this is often a late sign by which time neural enlargement should already be evident on MRI.

The role of hybrid CT-PET imaging is still being determined, but it may be useful in advanced disease for evaluation of nodal or distant metastasis and treatment response. The findings on CT-PET indicative of PNS include (a) linear or focal enhancement along the course of the cranial nerves and neural foramina, and (b) asymmetric fluorodeoxyglucose uptake in the pterygopalatine fossa, Meckel’s cave and cavernous sinus [70].

## Evaluation of regional and distant metastasis

PNI and PNS are risk factors for locoregional and distant metastasis. Patients with palpable lymph nodes can be referred for confirmatory fine needle aspiration or core biopsy, depending on institutional practice. If there is no palpable lymphadenopathy, the indications for nodal imaging are unclear. It should be noted that the false negative rate of clinical examination for regional nodal metastasis is 15–30% [71]. Therefore, in the presence of high-risk PNI parameters and other concerning histological and clinical risk factors (Tables 1 and 2), further imaging is warranted for the evaluation of subclinical nodal disease. Ultrasound in the hands of a skilled operator has been shown to have a greater sensitivity and specificity than CT and MRI for the detection of nodal metastases in head and neck SCC, (ultrasound: sens 87%, spec 86%; CT: sens 81%, spec 76%; and MRI: sens 81%, spec 63%) [72, 73]. Furthermore, ongoing ultrasound monitoring for early detection of metastatic nodal disease is preferable as it avoids repeated ionising radiation exposure. However, access to personnel and scanners will vary at each institution, so the exact protocols for evaluation of nodal metastasis should be discussed with a neuroradiologist and multidisciplinary team.

The role of sentinel lymph node biopsy (SLNB) in cSCC is ill-defined and there is no data to suggest that SLNB status affects recurrence or survival outcomes [71]. The NCCN guidelines are equivocal and do not incorporate SLNB into its treatment algorithms [47]. In periocular SCC with PNI, SLNB may be considered if there is significant concern due to multiple high-risk features (Tables 1 and 2), if there is no palpable lymphadenopathy and no radiological detection of locoregional metastasis [11].

## Prognosis

Both PNI and PNS are markers of an aggressive tumour profile. However, PNI portends a better prognosis than PNS and has comparatively better local control and cause-specific survival rates (local control 80% vs 55%; cause-specific survival 75% vs 65%, respectively) [74].

### Metastasis and disease-specific survival

In the periocular SCC literature, PNI positivity appears to be a strong predictor for regional and distant metastases, though these are small studies with insufficient data on long-term outcomes. In Faustina et al.’s series, 55.6% of PNI-positive tumours (5/9 patients) developed regional nodal metastasis and 22.2% (2/9 patients) had distant metastasis, despite treatment with wide local excision and postoperative radiotherapy [11]. In McNab et al.’s series of perineural orbital spread from cSCC, two-thirds of the cohort succumbed to disease-related mortality [57].

The association between PNI, recurrence and disease-specific death can be extrapolated from the head and neck cSCC literature [75, 76]. The incidence of PNI was significantly higher in lesions that recurred compared to recurrence-free lesions (56% vs 11%, respectively), and the relative risk (RR) of local recurrence in the presence of PNI was 4.30 (95% CI 2.80–6.60) [50, 77]. PNI was an independent predictor of nodal metastasis (RR 2.95, 95% CI 2.31–3.75), after adjusting for tumour location, differentiation pattern and depth of nerve involvement [50, 78]. In advanced head and neck cSCC, PNI was associated with worse disease-free survival at 2 years (54% PNI-positive vs 78% PNI-negative) and overall survival at 5 years (45% PNI-positive vs 76% PNI-negative) [77, 79, 80].

## Management of PNI

The periocular region is an inherently high-risk location and PNI is not always apparent in a preoperative biopsy. Therefore, for all periocular SCC lesions, we recommend surgical excision with intraoperative margin control in the first instance. For lesions without orbital invasion, en face margin control techniques such as Mohs micrographic surgery (MMS) or complete circumferential peripheral and deep margin assessment (CCPDMA), are preferred for establishing true histological clearance. In the Australian Mohs database study of periocular SCC, all cases with PNI (4.3% of the cohort) were treated with MMS alone and experienced no recurrence following 4–6 years of follow-up [8].

In the presence of radiologically confirmed orbital invasion, our preference is to perform CCPDMA with fast-track paraffin sections and delayed reconstruction, as appropriate orientation of frozen sections of orbital fat can be difficult. In the context of large tumours with orbital invasion, peripheral margin assessment may be difficult and standard fine breadloaf sections may be utilised. If the resection specimen demonstrates very high risk PNI features or if there are other multiple concurrent high-risk factors, then a multidisciplinary consult should be sought for consideration of adjuvant radiotherapy.

## Management of PNS

PNS to the orbit presents a unique challenge for achieving surgical clearance due to the potential for subclinical disease involving surrounding neurovascular structures [81]. Anatomically, Panizza et al.’s zonal classification of PNS in head and neck cSCC has implications for treatment options, in which Zone 1 and 2 disease is amenable to surgical resection to the zonal boundary [36].

- Zone 1: ophthalmic nerve (V_1_) to superior orbital fissure, infraorbital nerve (V_2_) to foramen rotundum, mandibular nerve (V_3_) to foramen ovale, facial nerve to stylomastoid foramen.
- Zone 2: trigeminal nerve branches, from Zone 1 to trigeminal ganglion; facial nerve: from Zone 1 to lateral end of internal auditory canal including geniculate ganglion.
- Zone 3: all nerves: proximal to the ganglion, into the brainstem.

### Zone 1 disease

There is a paucity of robust evidence to compare outcomes of exenteration versus globe-sparing techniques for Zone 1 disease. At our institution, for disease confined to the involved nerve with no significant orbital mass or soft tissue extension, our preference is for globe-sparing en bloc resection of the involved nerve up to its posterior margin. The transorbital endoscopic approach provides excellent visualisation of the involved structures and an opportunity to resect all nerve branch points. The frontal nerve can be stripped to the superior orbital fissure. Similarly for Zone 1 disease localised to the infraorbital nerve, it is possible to resect the nerve to the foramen rotundum via a transorbital route.

If there is significant tumour spread into the orbital soft tissue outside of the involved nerve, then exenteration to the apex is performed. Frozen section analysis of the apical tissues or posterior nerve margin may be undertaken and if positive, consideration can be given to an apical drillout to expose the lateral wall of the cavernous sinus and resect the ophthalmic nerve more posteriorly.

### Zone 2 disease

For Zone 2 disease, orbital exenteration with resection of the relevant ganglion is appropriate. Although there have only been small cohorts of ≤50 patients reported with such extensive disease, surgical resection combined with postoperative radiotherapy can achieve feasible long-term survival outcomes, with 5-year disease-specific survival of 64–75% [36, 82]. A large cohort of 120 patients from Warren and Panizza’s database in Queensland, Australia found that the 5-year disease-specific survival for Zone 1, 2 and 3 disease was 84%, 64% and 15%, respectively (*p* < 0.0001) [56].

### Zone 3 disease

Aggressive surgical resection is often deemed unsuitable for Zone 3 disease due its inherent association with lower survival likelihood and the risk of significant perioperative morbidity [36, 83].

#### Radiotherapy for PNI and PNS

For periocular SCC, some authors recommend adjuvant radiotherapy for all lesions with PNI or PNS [9, 57]. The rationale for this aggressive approach is that if recurrent disease were to occur in a major nerve trunk, it would inevitably have a poor prognosis and salvage therapy would incur significant morbidity. However, these were small retrospective studies of periocular SCC and the risk of visual compromise from radiotherapy must be considered. The average dose of adjuvant radiotherapy for periocular skin carcinomas is 50–60 Gy [5]. At these levels, severe dry eye, radiation keratopathy and cataract formation are common complications [84–86]. Overall, there is minimal discernible benefit to irradiate small-calibre PNI (<0.1 mm) alone. However, it is recommended if there are multiple high-risk features such as chronic immunosuppression, large tumour diameter >2 cm, poor differentiation, deep invasion beyond subcutaneous fat and lymphovascular invasion (Table 2) [44].

In the head and neck cSCC literature, PNS portends a poor prognosis and therefore combined resection and elective radiotherapy to the first echelon of regional lymph nodes has been advocated regardless of the margin status [53, 82]. However, the locoregional recurrence rate is variable, ranging from 41 to 62% [87, 88]. There is no standardised treatment protocol for postoperative radiotherapy, but a reasonable approach would be to include peripheral branches and the dermatomal distribution of the involved nerve [82]. The posterior extent of the radiotherapy field may vary based on whether the posterior surgical margin was positive, individual patient factors and institutional preferences. For instance, some may treat to 1 cm posterior to a negative surgical margin whilst others may extend further to the brainstem.

#### Chemotherapy

There is a paucity of data on the utility of chemotherapy in periocular or head and neck cSCC. Theoretically, chemotherapy should only have a limited role. Nerves affected by PNI undergo axonal degeneration and segmental infarction. As a result, blood supply to the perineural vascular plexuses is compromised, which inhibits the delivery of chemotoxins to tumour cells within the perineural space [89].

Individual case reports of multi-agent chemotherapy using methotrexate, bleomycin and cisplatin have found promising results for orbital perineural spread, but in the setting of progressive intracranial disease, there is no evidence of definitive improvement [33, 90]. It remains unclear if chemotherapy alone can achieve local control or if there is a synergistic role with radiation. Further studies are indicated to draw definitive conclusions.

#### Immunotherapy

Immune checkpoint inhibitors represent a novel treatment option for surgically unresectable cSCC with locally advanced or distant metastatic disease. In Migden et al.’s seminal paper on immunotherapy in metastatic cSCC, cemiplimab, a monoclonal antibody directed against the PD-1 receptor pathway, elicited a 50% response rate [91]. There is also emerging though limited data that neoadjuvant PD-1 inhibitor immunotherapy may be an option for locoregionally advanced and surgically resectable disease [92]. In a small pilot phase 2 trial, it was observed that neoadjuvant immunotherapy induced a substantial pathologic response such that it obviated the need for adjuvant radiotherapy [92].

In 2018, cemiplimab received approval from the US Federal Drug Administration to treat locally advanced or metastatic cSCC that did not meet criteria for curative surgical resection or curative radiotherapy. The following year, cemiplimab was approved by the European Commission and NICE advisory committees, the latter of which has made it available on the National Cancer Drugs Fund. In Australia, it is provisionally approved by the Therapeutic Goods Administration for similar candidates. The decisions for approval are largely due to encouraging results from a phase 2 single-arm trial with 44% of the cohort experiencing an objective clinical response and demonstrating a tolerable safety profile [93].

The evidence for immunotherapy in periocular SCC with PNI has been promising, but scant. There was one case report of a primary suprabrow SCC with orbital extension that recurred following radical surgical resection and radiotherapy, and was eventually amenable to cemiplimab [94]. A separate case series investigated the efficacy of pembrolizumab and cemiplimab for head and neck cSCC with perineural spread that was refractory to prior radical treatments [68]. Nine patients (82%) attained stabilisation or improved disease control, which compares favourably with the data from metastatic cSCC.

## Summary and recommendations for periocular SCC with PNI or PNS

### Risk stratification of PNI features

- High risk
  - Periocular region
  - Focal PNI (1–2 nerves on histological field)
  - Small-calibre nerve <0.1 mm
  - Dermal invasion only

- Very high risk
  - Named nerve involvement or large-calibre nerve ≥0.1 mm
  - Extensive PNI (>2 nerves on histological field)
  - Deep invasion beyond subcutaneous fat

### Imaging protocols for PNS

MRI is the recommended imaging modality for detecting PNS. The indications are as follows:

- Any symptoms of PNS
- Focal PNI with multiple high-risk histological or clinical features (Tables 1 and 2)
- Extensive PNI

The most useful imaging sequences are T1-weighted, T1WI fat-suppressed contrast-enhanced, and T2-weighted with fat saturation. The salient radiological signs of PNS are as follows:

- Nerve thickening or enlargement
- Nerve enhancement
- Obliteration of fat pads along the course of the involved nerve
- Erosion and widening of neural foramina
- Muscle denervation atrophy and adipogenic replacement

### Management

**Fig. 3:**
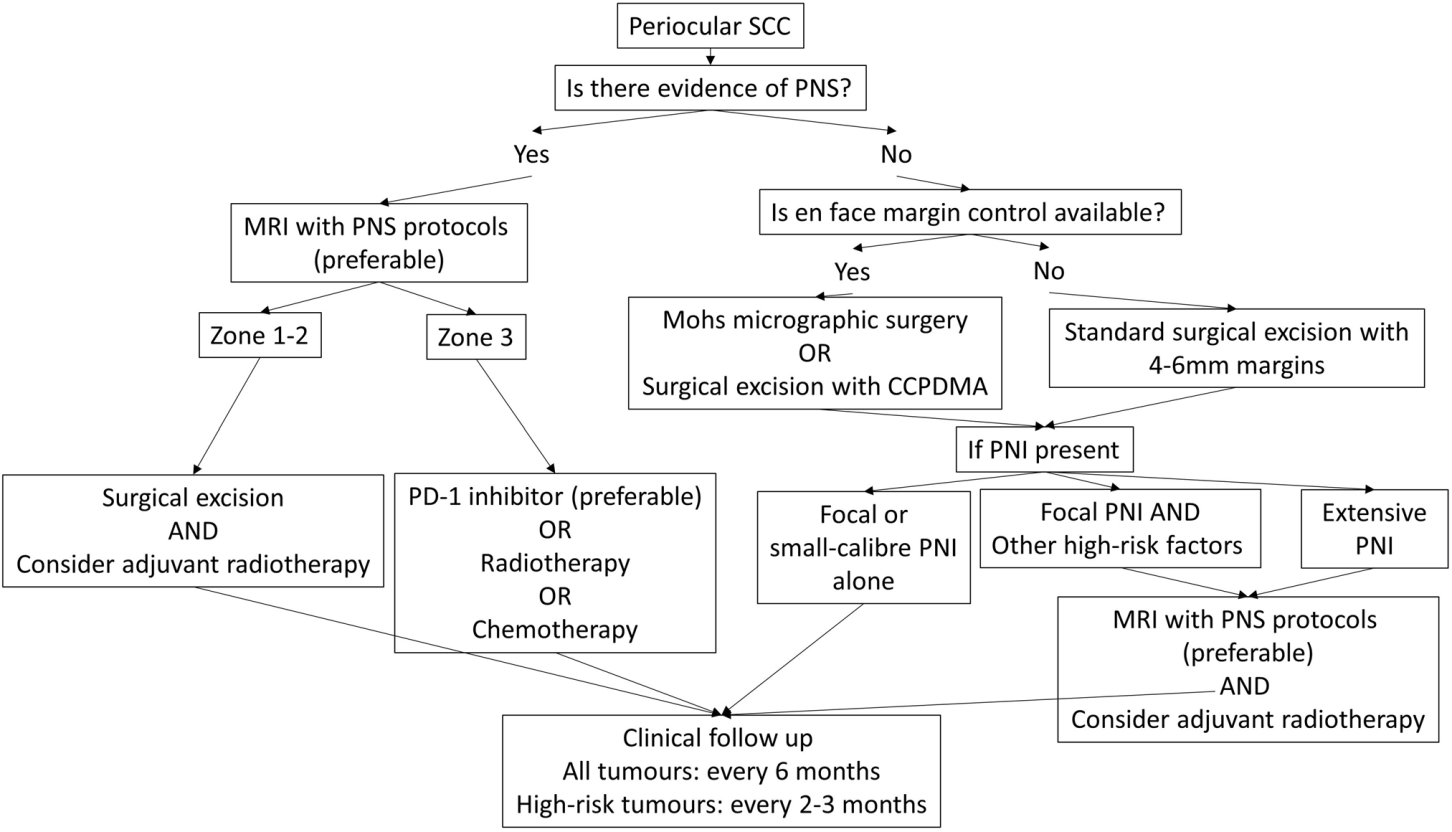
Management algorithm. The definition of high-risk factors encompasses both high-risk PNI parameters and high-risk clinical and histological tumour factors. High-risk PNI parameters include extratumoral involvement, large-calibre (≥0.1 mm diameter), named nerve, multiple nerves or deep invasion beyond subcutaneous fat. Other high-risk histological factors include poor differentiation, aggressive histological subtype and lymphovascular invasion. Additional high-risk clinical factors include chronic immunosuppression, tumour size larger than 2 cm and tumour thickness greater than 6 mm. CCPDMA complete circumferential peripheral and deep margin assessment via intraoperative frozen section or permanent sections with delayed reconstruction, MRI magnetic resonance imaging, PD-1 programmed cell death protein 1, PNI perineural invasion, PNS perineural spread, SCC squamous cell carcinoma.

A flowchart demonstrating our management algorithm is outlined in Fig. 3. For periocular SCC, we recommend all surgical candidates be offered en face margin controlled surgical excision if available. In the absence of orbital invasion, MMS is preferable, but CCPDMA via frozen section, or standard excision with a 4–6 mm margin and delayed reconstruction are reasonable alternatives. For lesions with only focal small-calibre PNI and no other high-risk features, regular clinical and imaging surveillance will suffice. Tumours with very high risk PNI histological features and multiple concurrent high-risk tumour factors (Tables 1 and 2) warrant multidisciplinary involvement to consider adjuvant radiotherapy.

All tumours with suspicion of PNS should undergo a 3T MRI with the protocols outlined in Table 4. Assessment of the draining regional nodes can also be considered, even in the absence of palpable lymphadenopathy. Such cases should ideally be discussed at a head and neck skin cancer multidisciplinary meeting. Zone 1 PNS isolated to the nerve can be addressed by a globe-sparing technique with stripping of the involved nerve to its posterior margin via the transorbital route. Zone 1 PNS with orbital soft tissue extension, or Zone 2 PNS, should undergo an exenteration and adjuvant radiotherapy. Zone 3 PNS is considered surgically unresectable disease, and may be eligible for radiotherapy, chemotherapy or PD-1 inhibitor immunotherapy depending on institutional protocols and availability [36].

## Follow-up

A vigilant follow-up schedule is an essential part of postoperative care for patients with periocular SCC and PNI, due to the risk of recurrence and perineural spread into the orbit and cranial fossa. It is estimated that 85% of recurrences occur within the first 2 years following initial treatment [30]. Routine ocular, skin and lymph node examinations should be performed every 6 months, while high-risk tumours warrant a shorter follow-up interval of 2–3 months. MRI surveillance of the orbit is recommended every 6 months for at least 5 years, then annually for life [95, 96]. Neck node surveillance can be performed by MRI or ultrasound, with the latter modality demonstrating a greater predictive accuracy in the hands of experienced sonographers [72, 73]. MRI is useful for delineating subtle soft tissue and perineural changes in situations where sensory symptoms are rendered unreliable from prior nerve resections. The interval for clinical and radiographic surveillance should be individualised in the setting of multiple high-risk histological features and/or comorbidities.

## Conclusion

Perineural invasion is an established adverse prognostic marker which confers a higher risk of local recurrence and regional and distant metastasis. A thorough clinical evaluation of periocular SCC with PNI should include risk stratification of PNI parameters and MRI with PNI protocols to detect perineural spread. Surgical excision with intraoperative en face margin control is strongly encouraged. Adjuvant radiotherapy for high-risk lesions improves the cure rate, but its utility in the periocular region should be balanced by visual morbidity risk. PD-1 inhibitor immunotherapy is suitable for non-surgical candidates with locally advanced or distant metastatic disease. Further studies, particularly randomised controlled trials, are necessary for the development of evidence-based oncology protocols.
